# *N*′-[6-(3,5-Dimethylpyrazol-1-yl)-1,2,4,5-tetrazin-3-yl]propanohydrazide

**DOI:** 10.1107/S1600536810041528

**Published:** 2010-10-23

**Authors:** Qi-dong Yan, Feng Xu, Jun Xu, Jian-jun Chen

**Affiliations:** a Department of Biological & Chemical Engineering, Taizhou Vocational & Technical College, Taizhou, 318000, People’s Republic of China

## Abstract

In the title compound, C_10_H_14_N_8_O, the tetra­zine and pyrazole rings form a dihedral angle of 48.81 (2)°. In the crystal, inter­molecular N—H⋯N and N—H⋯O hydrogen bonds link the mol­ecules into layers parallel to (101).

## Related literature

For related structures, see: Hu *et al.* (2004 ▶); Xu *et al.* (2010 ▶). For applications of 1,2,4,5-tetra­zine derivatives, see: Sauer (1996 ▶).
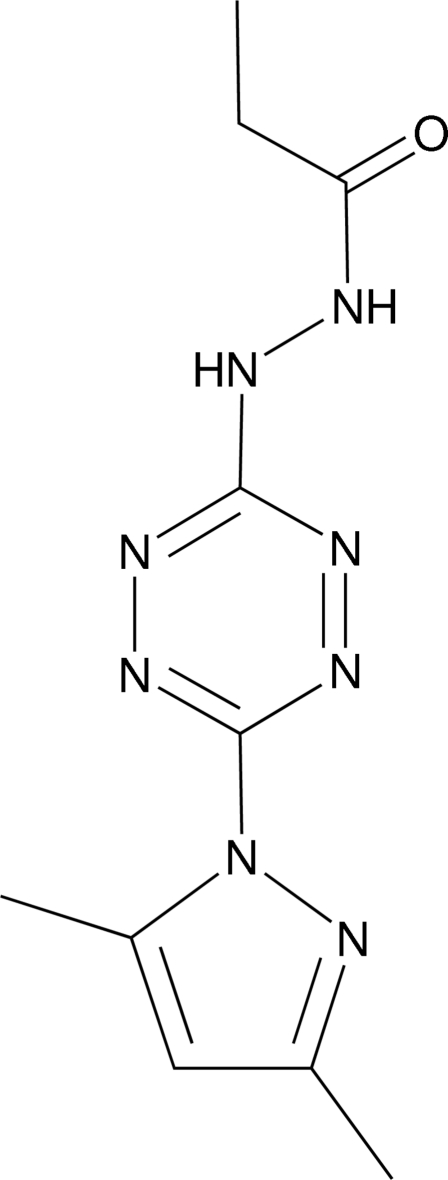


## Experimental

### 

#### Crystal data

C_10_H_14_N_8_O
*M* *_r_* = 262.29
Monoclinic, 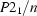

*a* = 10.896 (3) Å
*b* = 8.0354 (18) Å
*c* = 14.805 (3) Å
β = 101.243 (3)°
*V* = 1271.3 (5) Å^3^
*Z* = 4
Mo *K*α radiation
μ = 0.10 mm^−1^
*T* = 103 K
0.43 × 0.43 × 0.40 mm

#### Data collection

Rigaku AFC10/Saturn724+ diffractometer
11006 measured reflections
2889 independent reflections
2449 reflections with *I* > 2σ(*I*)
*R*_int_ = 0.026

#### Refinement

*R*[*F* ^2^ > 2σ(*F* ^2^)] = 0.036
*wR*(*F* ^2^) = 0.097
*S* = 1.00
2889 reflections
183 parameters
H atoms treated by a mixture of independent and constrained refinement
Δρ_max_ = 0.25 e Å^−3^
Δρ_min_ = −0.21 e Å^−3^

### 

Data collection: *CrystalClear* (Rigaku/MSC, 2008 ▶); cell refinement: *CrystalClear*; data reduction: *CrystalClear*; program(s) used to solve structure: *SHELXS97* (Sheldrick, 2008 ▶); program(s) used to refine structure: *SHELXL97* (Sheldrick, 2008 ▶); molecular graphics: *PLATON* (Spek, 2009 ▶); software used to prepare material for publication: *SHELXL97*.

## Supplementary Material

Crystal structure: contains datablocks global, I. DOI: 10.1107/S1600536810041528/cv2774sup1.cif

Structure factors: contains datablocks I. DOI: 10.1107/S1600536810041528/cv2774Isup2.hkl

Additional supplementary materials:  crystallographic information; 3D view; checkCIF report

## Figures and Tables

**Table 1:** Hydrogen-bond geometry (Å, °)

*D*—H⋯*A*	*D*—H	H⋯*A*	*D*⋯*A*	*D*—H⋯*A*
N14—H14*N*⋯O17^i^	0.859 (17)	1.980 (17)	2.821 (2)	166 (2)
N15—H15*N*⋯N8^ii^	0.893 (18)	1.996 (18)	2.882 (2)	171 (2)

Symmetry codes: (i) 
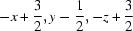
; (ii) 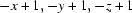.

**Fig. 1.**
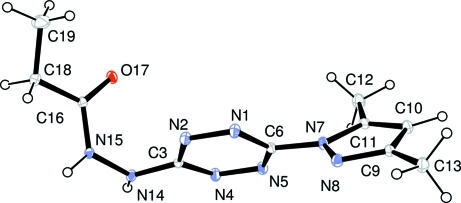
The structure of (I), shown with 30% probability displacement ellipsoids.

**Fig. 2.**
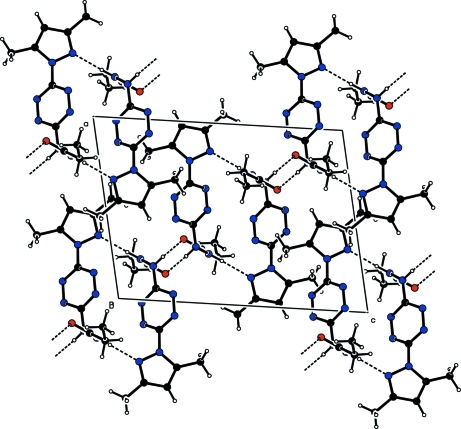
A portion of the crystal packing of (I), viewed down the b axis (N—H···O and N—H···N hydrogen bonds shown as dashed lines).

## supplementary crystallographic information

### Comment

1,2,4,5-Tetrazine derivatives have high potential for biological activity, possessing a wide spectrum of antiviral and antitumor properties. They have been widely used in pesticides and herbicides (Sauer,1996). In continuation of our study of the structure-activity relationships of 1,2,4,5-tetrazine derivatives (Hu *et al.*, 2004; Xu *et al.*, 2010), we present here the crystal structure of the title compound (I).

In (I) (Fig. 1), the essentially planar tetrazine ring forms a dihedral angle of 48.81 (2)° with the pyrazole ring. The N14/N15/C16/O17 and C16/C18/C19 planes form dihedral angles of 79.07 (2)° and 53.51 (2)°, respectively, with the tetrazine ring. In the crystal structure, intermolecular N—H···N and N—H···O hydrogen bonds (Table 1) link the molecules into layers parallel to the plane (101) (Fig. 2).

### Experimental

3,6-Di(3,5-dimethyl-1*H*-pyrazol-1-yl)-1,2,4,5-tetrazine (3.0 mmol), chloroform (10 ml) and pyridine(0.25 ml,3.1 mmol) were mixed. Propionyl chloride(3.0 mmol) in chloroform (10 ml) was added dropwise with stirring at room temperature. After the starting 1,2,4,5-tetrazine was completely consumed (the reaction courses was monitored by TLC, ethyl acetate system), evaporation of the chloroform, crude product was obtained and purified by preparative thin-layer chromatography over silica gel GF254(2 mm) (dichloromethane: petroleum ether=1:1). The solution of the compound in anhydrous ethanol was concentrated gradually at room temperature to afford single crystals, which was suitable for X-ray diffraction.

### Refinement

C-bound H atoms were placed in calculated positions with  C—H = 0.93 (aromatic) and 0.96 Å (methyl), and refined as riding, with *U*_iso_(H) = 1.2*U*_eq_(C). Amino H atoms were located on a difference map and isotropically refined.

### Crystal data

**Table:**

C_10_H_14_N_8_O	*F*(000) = 552
*M**_r_* = 262.29	*D*_x_ = 1.370 Mg m^−^^3^
Monoclinic, *P*2_1_/*n*	Mo *K*α radiation, λ = 0.71073 Å
Hall symbol: -P 2yn	Cell parameters from 3481 reflections
*a* = 10.896 (3) Å	θ = 3.1–27.5°
*b* = 8.0354 (18) Å	µ = 0.10 mm^−^^1^
*c* = 14.805 (3) Å	*T* = 103 K
β = 101.243 (3)°	Block, red
*V* = 1271.3 (5) Å^3^	0.43 × 0.43 × 0.40 mm
*Z* = 4	

### Data collection

**Table:**

Rigaku AFC10/Saturn724+ diffractometer	2449 reflections with *I* > 2σ(*I*)
Radiation source: Rotating Anode	*R*_int_ = 0.026
graphite	θ_max_ = 27.5°, θ_min_ = 3.2°
Detector resolution: 28.5714 pixels mm^-1^	*h* = −14→14
phi and ω scans	*k* = −10→9
11006 measured reflections	*l* = −19→15
2889 independent reflections	

### Refinement

**Table:**

Refinement on *F*^2^	Primary atom site location: structure-invariant direct methods
Least-squares matrix: full	Secondary atom site location: difference Fourier map
*R*[*F*^2^ > 2σ(*F*^2^)] = 0.036	Hydrogen site location: inferred from neighbouring sites
*wR*(*F*^2^) = 0.097	H atoms treated by a mixture of independent and constrained refinement
*S* = 1.00	*w* = 1/[σ^2^(*F*_o_^2^) + (0.0536*P*)^2^ + 0.356*P*] where *P* = (*F*_o_^2^ + 2*F*_c_^2^)/3
2889 reflections	(Δ/σ)_max_ = 0.001
183 parameters	Δρ_max_ = 0.25 e Å^−^^3^
0 restraints	Δρ_min_ = −0.21 e Å^−^^3^

### Special details

**Table:**

Geometry. All e.s.d.'s (except the e.s.d. in the dihedral angle between two l.s. planes) are estimated using the full covariance matrix. The cell e.s.d.'s are taken into account individually in the estimation of e.s.d.'s in distances, angles and torsion angles; correlations between e.s.d.'s in cell parameters are only used when they are defined by crystal symmetry. An approximate (isotropic) treatment of cell e.s.d.'s is used for estimating e.s.d.'s involving l.s. planes.
Refinement. Refinement of *F*^2^ against ALL reflections. The weighted *R*-factor *wR* and goodness of fit *S* are based on *F*^2^, conventional *R*-factors *R* are based on *F*, with *F* set to zero for negative *F*^2^. The threshold expression of *F*^2^ > σ(*F*^2^) is used only for calculating *R*-factors(gt) *etc*. and is not relevant to the choice of reflections for refinement. *R*-factors based on *F*^2^ are statistically about twice as large as those based on *F*, and *R*- factors based on ALL data will be even larger.

### Fractional atomic coordinates and isotropic or equivalent isotropic displacement parameters (Å^2^)

**Table:**

	*x*	*y*	*z*	*U*_iso_*/*U*_eq_	
O17	0.65146 (8)	0.86367 (11)	0.70314 (6)	0.0179 (2)	
N1	0.36485 (9)	0.55187 (13)	0.58619 (7)	0.0181 (2)	
N2	0.48288 (9)	0.59455 (13)	0.59073 (7)	0.0178 (2)	
N4	0.54093 (9)	0.41800 (13)	0.72319 (7)	0.0178 (2)	
N5	0.42231 (9)	0.37460 (13)	0.71646 (7)	0.0180 (2)	
N7	0.21537 (9)	0.38630 (13)	0.63454 (7)	0.0153 (2)	
N8	0.15710 (9)	0.34258 (13)	0.54720 (7)	0.0162 (2)	
N14	0.69045 (9)	0.54690 (13)	0.65843 (7)	0.0150 (2)	
N15	0.72507 (9)	0.68179 (12)	0.61051 (7)	0.0143 (2)	
C3	0.56793 (11)	0.51973 (14)	0.65674 (8)	0.0141 (2)	
C6	0.34064 (11)	0.43941 (15)	0.64680 (8)	0.0144 (2)	
C9	0.04184 (11)	0.30101 (15)	0.55470 (9)	0.0160 (3)	
C10	0.02513 (11)	0.31991 (16)	0.64640 (9)	0.0182 (3)	
H10	−0.0491	0.2986	0.6691	0.022*	
C11	0.13712 (11)	0.37494 (15)	0.69613 (8)	0.0162 (3)	
C12	0.17476 (12)	0.42513 (18)	0.79463 (9)	0.0225 (3)	
H12A	0.1010	0.4254	0.8234	0.027*	
H12B	0.2114	0.5369	0.7983	0.027*	
H12C	0.2366	0.3461	0.8269	0.027*	
C13	−0.04989 (11)	0.24561 (17)	0.47157 (9)	0.0202 (3)	
H13A	−0.0168	0.2705	0.4161	0.024*	
H13B	−0.1293	0.3045	0.4689	0.024*	
H13C	−0.0637	0.1255	0.4752	0.024*	
C16	0.70060 (10)	0.83713 (14)	0.63648 (8)	0.0138 (2)	
C18	0.73811 (12)	0.97462 (16)	0.57793 (9)	0.0204 (3)	
H18A	0.8278	1.0008	0.5999	0.024*	
H18B	0.7275	0.9354	0.5135	0.024*	
C19	0.66242 (18)	1.13003 (19)	0.58044 (13)	0.0396 (4)	
H19A	0.5744	1.1071	0.5539	0.048*	
H19B	0.6936	1.2175	0.5447	0.048*	
H19C	0.6698	1.1669	0.6444	0.048*	
H14N	0.7449 (16)	0.507 (2)	0.7030 (12)	0.032 (5)*	
H15N	0.7596 (16)	0.663 (2)	0.5615 (12)	0.032 (4)*	

### Atomic displacement parameters (Å^2^)

**Table:**

	*U*^11^	*U*^22^	*U*^33^	*U*^12^	*U*^13^	*U*^23^
O17	0.0174 (4)	0.0197 (5)	0.0189 (4)	−0.0030 (3)	0.0092 (3)	−0.0036 (4)
N1	0.0153 (5)	0.0192 (5)	0.0195 (5)	−0.0025 (4)	0.0025 (4)	0.0026 (4)
N2	0.0153 (5)	0.0183 (5)	0.0196 (5)	−0.0022 (4)	0.0025 (4)	0.0032 (4)
N4	0.0142 (5)	0.0190 (5)	0.0207 (5)	−0.0005 (4)	0.0045 (4)	0.0042 (4)
N5	0.0161 (5)	0.0184 (5)	0.0203 (5)	−0.0011 (4)	0.0057 (4)	0.0033 (4)
N7	0.0141 (5)	0.0190 (5)	0.0133 (5)	−0.0019 (4)	0.0040 (4)	−0.0007 (4)
N8	0.0158 (5)	0.0187 (5)	0.0147 (5)	−0.0018 (4)	0.0042 (4)	−0.0018 (4)
N14	0.0139 (5)	0.0135 (5)	0.0177 (5)	−0.0003 (4)	0.0034 (4)	0.0048 (4)
N15	0.0170 (5)	0.0132 (5)	0.0146 (5)	−0.0019 (4)	0.0077 (4)	0.0014 (4)
C3	0.0164 (5)	0.0115 (5)	0.0151 (6)	−0.0001 (4)	0.0051 (4)	−0.0015 (4)
C6	0.0138 (5)	0.0148 (6)	0.0156 (6)	−0.0012 (4)	0.0050 (4)	−0.0013 (4)
C9	0.0143 (5)	0.0151 (6)	0.0194 (6)	−0.0003 (4)	0.0054 (5)	0.0012 (5)
C10	0.0162 (5)	0.0200 (6)	0.0202 (6)	−0.0010 (5)	0.0077 (5)	0.0011 (5)
C11	0.0172 (6)	0.0158 (6)	0.0177 (6)	0.0010 (4)	0.0084 (5)	0.0024 (5)
C12	0.0227 (6)	0.0285 (7)	0.0178 (6)	−0.0022 (5)	0.0075 (5)	−0.0005 (5)
C13	0.0171 (6)	0.0221 (6)	0.0217 (6)	−0.0034 (5)	0.0042 (5)	−0.0009 (5)
C16	0.0105 (5)	0.0148 (6)	0.0160 (6)	−0.0013 (4)	0.0026 (4)	0.0000 (4)
C18	0.0224 (6)	0.0167 (6)	0.0241 (7)	−0.0016 (5)	0.0096 (5)	0.0034 (5)
C19	0.0561 (10)	0.0238 (8)	0.0457 (10)	0.0138 (7)	0.0269 (8)	0.0154 (7)

### Geometric parameters (Å, °)

**Table:**

O17—C16	1.2297 (15)	C9—C13	1.4940 (17)
N1—N2	1.3200 (14)	C10—C11	1.3698 (17)
N1—C6	1.3355 (16)	C10—H10	0.9500
N2—C3	1.3499 (16)	C11—C12	1.4914 (18)
N4—N5	1.3235 (14)	C12—H12A	0.9800
N4—C3	1.3547 (16)	C12—H12B	0.9800
N5—C6	1.3300 (16)	C12—H12C	0.9800
N7—C11	1.3675 (15)	C13—H13A	0.9800
N7—N8	1.3705 (14)	C13—H13B	0.9800
N7—C6	1.4076 (15)	C13—H13C	0.9800
N8—C9	1.3249 (15)	C16—C18	1.5091 (17)
N14—C3	1.3480 (15)	C18—C19	1.501 (2)
N14—N15	1.3874 (14)	C18—H18A	0.9900
N14—H14N	0.860 (18)	C18—H18B	0.9900
N15—C16	1.3480 (16)	C19—H19A	0.9800
N15—H15N	0.893 (19)	C19—H19B	0.9800
C9—C10	1.4128 (17)	C19—H19C	0.9800
			
N2—N1—C6	117.36 (10)	C10—C11—C12	131.24 (11)
N1—N2—C3	116.47 (10)	C11—C12—H12A	109.5
N5—N4—C3	116.84 (10)	C11—C12—H12B	109.5
N4—N5—C6	116.81 (10)	H12A—C12—H12B	109.5
C11—N7—N8	112.19 (10)	C11—C12—H12C	109.5
C11—N7—C6	130.48 (10)	H12A—C12—H12C	109.5
N8—N7—C6	117.30 (9)	H12B—C12—H12C	109.5
C9—N8—N7	104.95 (10)	C9—C13—H13A	109.5
C3—N14—N15	118.95 (10)	C9—C13—H13B	109.5
C3—N14—H14N	119.1 (12)	H13A—C13—H13B	109.5
N15—N14—H14N	118.1 (12)	C9—C13—H13C	109.5
C16—N15—N14	119.27 (10)	H13A—C13—H13C	109.5
C16—N15—H15N	122.0 (11)	H13B—C13—H13C	109.5
N14—N15—H15N	118.7 (11)	O17—C16—N15	122.06 (11)
N14—C3—N2	118.61 (11)	O17—C16—C18	122.90 (11)
N14—C3—N4	116.02 (10)	N15—C16—C18	115.04 (11)
N2—C3—N4	125.37 (11)	C19—C18—C16	112.53 (11)
N5—C6—N1	126.59 (11)	C19—C18—H18A	109.1
N5—C6—N7	117.82 (11)	C16—C18—H18A	109.1
N1—C6—N7	115.60 (10)	C19—C18—H18B	109.1
N8—C9—C10	110.81 (11)	C16—C18—H18B	109.1
N8—C9—C13	119.93 (11)	H18A—C18—H18B	107.8
C10—C9—C13	129.25 (11)	C18—C19—H19A	109.5
C11—C10—C9	106.44 (11)	C18—C19—H19B	109.5
C11—C10—H10	126.8	H19A—C19—H19B	109.5
C9—C10—H10	126.8	C18—C19—H19C	109.5
N7—C11—C10	105.61 (11)	H19A—C19—H19C	109.5
N7—C11—C12	123.07 (11)	H19B—C19—H19C	109.5
			
C6—N1—N2—C3	−0.04 (16)	C11—N7—C6—N1	−130.25 (13)
C3—N4—N5—C6	−2.11 (16)	N8—N7—C6—N1	47.37 (15)
C11—N7—N8—C9	−0.96 (13)	N7—N8—C9—C10	0.78 (13)
C6—N7—N8—C9	−179.01 (10)	N7—N8—C9—C13	−179.96 (10)
C3—N14—N15—C16	64.25 (15)	N8—C9—C10—C11	−0.34 (14)
N15—N14—C3—N2	17.35 (16)	C13—C9—C10—C11	−179.53 (12)
N15—N14—C3—N4	−162.40 (10)	N8—N7—C11—C10	0.75 (13)
N1—N2—C3—N14	173.64 (10)	C6—N7—C11—C10	178.47 (12)
N1—N2—C3—N4	−6.63 (18)	N8—N7—C11—C12	−176.21 (11)
N5—N4—C3—N14	−172.47 (10)	C6—N7—C11—C12	1.5 (2)
N5—N4—C3—N2	7.79 (18)	C9—C10—C11—N7	−0.25 (13)
N4—N5—C6—N1	−4.45 (19)	C9—C10—C11—C12	176.37 (13)
N4—N5—C6—N7	175.75 (10)	N14—N15—C16—O17	2.05 (17)
N2—N1—C6—N5	5.63 (19)	N14—N15—C16—C18	−178.04 (10)
N2—N1—C6—N7	−174.57 (10)	O17—C16—C18—C19	−25.57 (18)
C11—N7—C6—N5	49.57 (18)	N15—C16—C18—C19	154.53 (13)
N8—N7—C6—N5	−132.81 (12)		

### Hydrogen-bond geometry (Å, °)

**Table:**

*D*—H···*A*	*D*—H	H···*A*	*D*···*A*	*D*—H···*A*
N14—H14N···O17^i^	0.859 (17)	1.980 (17)	2.821 (2)	166 (2)
N15—H15N···N8^ii^	0.893 (18)	1.996 (18)	2.882 (2)	171 (2)

Symmetry codes: (i) −*x*+3/2, *y*−1/2, −*z*+3/2; (ii) −*x*+1, −*y*+1, −*z*+1.
